# Current Evidence on Optimal Intake of Animal-Source Foods and Health Status

**DOI:** 10.1016/j.advnut.2025.100466

**Published:** 2025-06-18

**Authors:** Lukas Schwingshackl, Sabrina Schlesinger

**Affiliations:** 1 Institute for Evidence in Medicine, Medical Center—University of Freiburg, Faculty of Medicine, University of Freiburg, Freiburg, Germany; 2 Institute for Biometrics and Epidemiology, German Diabetes Center, Leibniz Center for Diabetes Research at Heinrich Heine University Düsseldorf, Düsseldorf, Germany; 3 German Center for Diabetes Research (DZD e.V.), Partner Düsseldorf, München-Neuherberg, Germany

In the ongoing global debate about the role of animal-source foods (ASFs) in a healthy and sustainable diet, clarity is urgently needed. ASF such as meat, poultry, fish, eggs, and dairy are important sources of essential nutrients, including high-quality protein, iron, vitamin B-12, and zinc. At the same time, the consumption of ASF has been associated with an increased risk of noncommunicable diseases and adverse environmental impacts. The coexistence of nutritional benefits and potential health and environmental risks has contributed to ongoing scientific debate and has posed significant challenges to the development of evidence-based dietary guidelines [1]. To address the complexity and heterogeneity of the existing evidence on ASF and health outcomes, Rios-Leyvraz and Montez [2] conducted a scoping review commissioned by the WHO.

The objective of this review was to systematically map the current body of evidence on ASF intake and its associations with health-related outcomes across different population groups and life stages. Designed to inform the development of forthcoming WHO dietary guideline, the review synthesized >650 systematic, umbrella, and scoping reviews published between 2019 and 2024, encompassing over ∼500 cohort studies (>1600 publications) and ∼400 RCTs. Through this comprehensive synthesis, the authors identified topics extensively covered in the literature, including cardiovascular disease (CVD), cancer, and type 2 diabetes—and critical knowledge gaps, particularly concerning children, pregnant women, and populations in low-resource or food-insecure settings.

The findings revealed substantial variation in the amount, thematic focus, and consistency of the available evidence across various categories of ASFs. Dairy and fish were the most extensively examined food groups, with multiple reviews reporting on their associations with cardiometabolic health, bone health, and mortality. In contrast, poultry and eggs were less frequently addressed, and the evidence concerning specific subcategories—such as fermented dairy products like yogurt—was often limited.

The distribution of health outcomes assessed in the included reviews reflects, in part, the imbalance between observational and interventional study designs. A large proportion of the evidence base is derived from prospective cohort studies, which predominantly examined long-term outcomes such as CVD, cancer, type 2 diabetes, and all-cause mortality. These outcomes were particularly frequent in studies investigating red and processed meats. In contrast, RCTs primarily focused on intermediate disease markers, including blood lipids, blood pressure, glycemic control, anthropometric outcomes, and inflammatory markers. Moreover, for several health outcomes of high public health relevance—such as bone health, cognitive function, mental health, pregnancy outcomes, and child development—the available evidence was scarce, highlighting an important gap in the literature.

The review provides also a comprehensive overview of the analytical approaches used across existing evidence syntheses to evaluate associations between ASF intake and health outcomes. Most reviews conducted meta-analysis on high-intake and low-intake comparisons, a common strategy in nutritional epidemiology that facilitates comparability across studies. In addition, many reviews conducted dose–response analyses, allowing for a more nuanced understanding of the shape and strength of associations across intake levels. These approaches were particularly informative in the context of red and processed meat, where positive associations with CVD, cancer, mortality, and type 2 diabetes were frequently observed. Although informative, these approaches do not reflect real-world dietary pattern, where foods are typically substituted rather than simply increased or decreased in isolation. Substitution models address this limitation by assessing the comparative associations of replacing one food with another [3]. However, such analyses were underrepresented in the included reviews. Recent pairwise meta-analyses based on substitutions in prospective cohort studies—such as those by Neuenschwander et al. [4], Kiesswetter et al. [5], and Papp et al. [6]—provide important insights into the potential benefits of replacing ASF with plant-based foods such as legumes, nuts, or whole grains. These studies consistently report lower risks of CVD, type 2 diabetes, and mortality, although the certainty of evidence ranged from moderate to very low.

To address the limitations of pairwise comparisons, substitutions of dietary factors can be integrated into network meta-analysis (NMA) frameworks [7]. So far, this approach has been used for macronutrients, for example, NMAs of RCTs have been conducted to compare isocaloric exchanges of different dietary sugars [8] or fats [9]. More recently, Wallerer et al. [10] extended the application of NMA to observational studies, modeling multiple nutrient-based substitutions simultaneously in relation to all-cause mortality. These developments highlight the potential of NMA-based substitution models to provide a robust framework for evaluating complex dietary substitutions. Nevertheless, substitution models—particularly those based on observational data—are subject to methodological challenges, including residual confounding, heterogenous comparator definitions, and assumptions about energy equivalence [3].

The scoping review by Rios-Leyvraz and Montez [2] offers a timely and methodologically rigorous synthesis of the existing evidence on ASF intake and health outcomes. Among its key strengths are the broad scope of included literature, the structured mapping across population groups and life stages, and the clear identification of research gaps—particularly for vulnerable populations such as children, pregnant women, and individuals in low-resource settings. In addition, the review provides valuable insights into the diversity of study designs and analytical approaches used of the research landscape. However, the absence of a systematic quality appraisal limits the ability to critically evaluate the strength and consistency of the underlying evidence. Moreover, the review did not stratify the synthesis along key analytical dimensions, such as dietary pattern, quantitative intake levels [i.e., (linear compared with nonlinear) dose response], or the specific context of dietary substitutions—including the type of replacement foods.

The findings of this scoping review map the current research landscape on ASF and health outcomes, emphasizing the need for a more coherent, methodologically robust, and sustainability-informed research agenda. Future studies should move beyond simplistic exposure contrasts, incorporate substitution-based modeling, and reflect the needs of diverse populations. Only through such integrated approaches can dietary guidelines be both evidence-based and globally relevant.

## Author contributions

The authors’ responsibilities were as follows – LS, SS: drafted the manuscript; and both authors: read and approved the final version.

## Funding

The authors reported no funding received for this study.

## Conflict of interest

LS is an Editor for *Advances in Nutrition* and played no role in the Journal’s evaluation of the manuscript.
